# The Controllability of *Caenorhabditis elegans* Neural Network from Larva to Adult

**DOI:** 10.3390/biomimetics10110744

**Published:** 2025-11-05

**Authors:** Jian Liu, Peng Zhao, Gang Wang, Tao Fang, Ye Yuan

**Affiliations:** 1 Institute of Machine Intelligence, University of Shanghai for Science and Technology, Shanghai 200093, China; liujian92@usst.edu.cn (J.L.); gwang@usst.edu.cn (G.W.); 2 Shanghai Jiao Tong University, Shanghai 200240, China; zhaopeng_2017@alumni.sjtu.edu.cn (P.Z.); tfang@sjtu.edu.cn (T.F.)

**Keywords:** controllability, target control, neural development, neural network, *C. elegans*

## Abstract

Biological neural networks undergo dynamic structural and functional changes during development, yet how their controllability evolves across different life stages remains largely unexplored. Here, we investigate the neural network of *Caenorhabditis elegans* (*C. elegans*), a fully mapped model organism, to examine changes in network controllability from larval stages to adulthood. Using structural controllability and target control frameworks, we show that while global neural controllability progressively increases with developmental complexity, muscle-target controllability declines after early larval stages, indicating a functional shift in control priorities. Furthermore, a comparative analysis between hermaphroditic and male adults reveals that overall controllability remains similar despite substantial differences in neural architecture, with hermaphrodites exhibiting slightly higher efficiency. These findings highlight fundamental principles of how neural circuits reorganize during maturation and suggest that controllability analysis can provide valuable insights into neural function, sex-specific behaviors, and potential applications in modeling developmental and degenerative disorders.

## 1. Introduction

Understanding the developmental processes of biological neural systems is fundamental to uncovering the mechanisms that underlie complex behaviors and functional regulation [1]. Traditional experimental approaches, however, face substantial challenges: large-scale neural networks are difficult to map, resource-intensive to study, and rarely provide complete information on how connectivity evolves across development. To address these limitations, methods from control theory [2,3] and complex network science [2,4] offer powerful analytical tools to study how neural circuits form, reorganize, and become controllable during maturation. In particular, analyzing both global and target controllability provides insights into how network architecture supports functional behaviors and how disruptions may contribute to developmental or degenerative disorders.

Within this interdisciplinary framework, *Caenorhabditis elegans* (*C. elegans*) [5] stands out as an ideal model organism. Its connectome is fully mapped—302 neurons in hermaphrodites and 383 in males, interconnected by 7000 chemical and 2000 electrical synapses [6]—allowing unprecedented resolution in structural and functional analysis. Despite nonlinear neuronal dynamics [3], the *C. elegans* network can be effectively approximated by linear or simplified models [7], making it suitable for controllability analysis. Its short life cycle and transparent body [8] also enable direct experimental validation of theoretical predictions [9]. These advantages position *C. elegans* as a unique system for exploring the interplay between network structure, controllability, and behavior across development.Recent advances have provided a functional-level understanding of neural signal propagation in *C. elegans*, linking anatomical connectivity to the dynamics of information flow across the nervous system [10]. Such studies highlight the growing interest in bridging structural connectomics and functional control frameworks, which motivates our controllability-based analysis across developmental stages.

Several studies have applied network control theory to *C. elegans*. Liu et al. [11] established general controllability principles for complex networks, including *C. elegans*. Gao et al. [12] introduced target control methods applicable to neural circuits. Yan et al. [2] identified key control nodes within the *C. elegans* connectome, while Liu et al. [13,14,15] examined motif-based organization and developmental changes. Collectively, these works demonstrate the value of control-theoretic approaches, but most have focused on static or single-stage networks.

Despite these advances, a critical question remains unresolved: how does controllability evolve throughout neural development? While this question is difficult to address in higher organisms, *C. elegans* offers a rare opportunity, as its complete connectome has been reconstructed across larval and adult stages [16]. In this study, we systematically analyze the controllability of *C. elegans* from larva to adulthood, with particular attention to both global and target controllability. Furthermore, we compare adult hermaphrodites and males to reveal sex-specific differences in control properties. Our findings uncover fundamental principles of how neural circuits reorganize during maturation and highlight the potential of controllability analysis to inform studies of complex nervous systems, including the human brain [17].

## 2. Materials and Methods

### 2.1. *C. elegans* Neural Network Dynamics

Although the nervous system of *C. elegans* is relatively simple, it exhibits complex neural signal activities primarily based on graded potentials [18]. Unlike the all-or-nothing action potentials observed in vertebrates, graded potentials are continuous changes in membrane potential that reflect the degree of neuronal excitation. Their magnitude varies with stimulus intensity, and in chemical synaptic transmission, neurotransmitters bind to postsynaptic receptors, opening ion channels and thereby altering the membrane potential.

The dynamics of nematode neurons can be described by nonlinear membrane potential models. For example, Hasani et al. [19] and Lechner et al. [20] introduced the following formulation:

(1) $${\dot{x}}_{i}=f\;\left(x_{i}\right)=-\left(\frac{1}{\tau_{i}}+\frac{w_{ij}}{C_{m_{i}}}\;\sigma_{i}\left(x_{j}\right)\right)x_{i}+\left(\frac{x_{{\mathrm{leak}}_{i}}}{\tau_{i}}+\frac{w_{ij}}{C_{m_{i}}}\;\sigma_{i}\left(x_{j}\right)E_{ij}\right)$$

where $\tau_{i}$ is the time constant of neuron *i*, $w_{ij}$ is the synaptic weight from neuron *i* to neuron *j*, $C_{m_{i}}$ is the membrane capacitance, and $\sigma_{i}\left(x_{j}\right)$ is the activation function defined as

(2) $$\sigma_{i}\left(x_{j}\right)=\frac{1}{1+e^{-\gamma_{ij}\left(x_{j}-\mu_{ij}\right)}}.$$

Here, $x_{{\mathrm{leak}}_{i}}$ denotes the resting potential, and $E_{ij}$ is the reversal synaptic potential, which determines the polarity of the synapse. Other studies, such as Izquierdo [21] and Liu et al. [15], have adopted alternative nonlinear formulations to capture the hybrid dynamics of electrical and chemical synapses.

While such nonlinear models provide biological realism, their high dimensionality and nonlinear coupling make large-scale analysis intractable. To facilitate network-level studies, simplified linear approximations are often employed. By treating each neuron as a node and each synapse as an edge, the neural network can be expressed in the standard state–space form:

(3) $$\dot{x}=Ax+Bu,$$

where *A* is the connectivity matrix of synaptic connections and *B* represents external inputs.

When considering muscles and other effectors as outputs of the system, the model extends to

(4) $$\left\{\begin{array}{c} \dot{x}=Ax+Bu,\\ y=Cx, \end{array}\right.$$

where *C* is the output matrix.

This linear representation captures the essential topology of the network while providing a tractable framework for controllability analysis. Such a formulation not only reduces the complexity of the underlying nonlinear dynamics but also enables a systematic evaluation of how control signals can influence network behavior—a perspective central to the concept of controllability.

### 2.2. Controllability of Complex Network and Neural Network

#### 2.2.1. Controllability

Controllability is a fundamental concept in control theory, describing the ability of a system to reach any desired state from any initial state within finite time through appropriate inputs [11,22,23]. In the context of neural networks, controllability reflects the capacity to regulate neuronal activity and network states via external stimulation or internal modulation.

For a linear system of *N* nodes as given in Equation (3), controllability can be evaluated by the Kalman rank condition:

(5) $$R=\left[\;B\;\;AB\;\;A^{2}B\;\;\cdots \;\;A^{\;n-1}B\;\right],$$

where *A* is the connectivity matrix and *B* is the input matrix. If

rank(R)=N,

the system is controllable [11].

In practice, however, applying this criterion directly to complex networks is often infeasible. First, precise connection weights are rarely available, making *A* and *B* uncertain. Second, the large scale of biological networks renders matrix computations computationally demanding and numerically unstable. To overcome these challenges, attention shifts from exact controllability to structural controllability, which depends only on the network topology—that is, the positions of nonzero elements in *A* and *B*—rather than their actual values. If the structural pattern satisfies the Kalman condition, the network is considered structurally controllable.

Even so, verifying the rank criterion at large scale remains challenging. An equivalent graph-theoretic formulation provides a more tractable approach [11]. By mapping network states to nodes and interactions to edges, structural controllability reduces to a maximum matching problem on the graph. The unmatched nodes in this maximum matching are the driver nodes, which must be directly controlled to ensure global controllability.

For a network of *N* nodes, let $N_{d}$ denote the number of driver nodes. Since network size varies, we use the normalized measure

(6) $$n_{d}=\frac{N_{d}}{N},$$

where a smaller $n_{d}$ indicates higher controllability.

**Figure 1:**
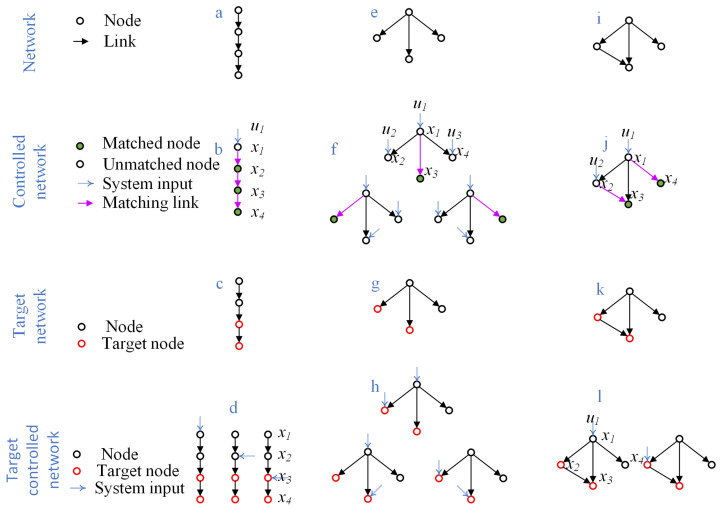
Control and target control of simple networks.

Illustrative examples are shown in Figure 1. In a directed path of four nodes (Figure 1a,b), only the first node is unmatched, giving $N_{d}=1$ and $n_{d}=\frac{1}{4}=0.25$, which represents high controllability. By contrast, in a star network of the same size (Figure 1c,d), three nodes remain unmatched, giving $N_{d}=3$ and $n_{d}=\frac{3}{4}=0.75$, indicating low controllability. An intermediate case (Figure 1e,f) yields $N_{d}=2$ and $n_{d}=\frac{2}{4}=0.5$. These examples demonstrate how network topology fundamentally shapes controllability, independent of exact synaptic strengths.

#### 2.2.2. Target Control

In many practical scenarios, it is not necessary to control all nodes of a network. Instead, the goal may be to regulate a subset of key outputs, referred to as target nodes. This approach, known as target control, seeks to drive selected outputs to desired states through suitable control inputs, without requiring full control of the entire system. By focusing on critical functional nodes—such as muscles in biological neural networks—target control reduces both the computational complexity and the cost of intervention, while retaining practical effectiveness.

For the linear system (4), output controllability can be evaluated by the so-called output controllability matrix:

(7) $$R_{\mathrm{out}}=\left[\;CB\;\;CAB\;\;CA^{2}B\;\;\cdots \;\;CA^{\;n-1}B\;\right],$$

where *C* is the output matrix. If

$$\mathrm{rank}(R_{\mathrm{out}})=m,$$

with *m* the dimension of the output vector *y*, then the system is said to be output controllable. Similar to global controllability, this criterion becomes computationally intractable for large-scale nonlinear systems, motivating the use of structural output controllability, which depends only on the zero–nonzero pattern of *A*, *B*, and *C*.

Gao et al. [12] showed that multiple target nodes can sometimes be controlled by a single driver node, depending on the distances within the network. For example, in the chain network of Figure 1c, if nodes 3 and 4 are designated as targets, then controlling node 1, 2, or 3 suffices to drive both targets, yielding $N_{d}=1$ and $n_{d}=\frac{1}{4}=0.25$. In contrast, in the network of Figure 1g, if nodes 2 and 3 are targets, at least two driver nodes are required (Figure 1h). For global controllability of the same network, however, three nodes must be controlled (Figure 1f).

These examples highlight two general observations: (i) the number of driver nodes required for target controllability is always less than or equal to that for global controllability, since only a subset of outputs needs to be regulated; and (ii) unlike structural controllability, identifying driver nodes for target controllability cannot be reduced to a maximum matching problem, and thus is computationally more challenging.

#### 2.2.3. Control and Target Control for Neural Network

In biological neural systems, controllability refers to the capacity to alter neuronal activity and network states through external inputs such as electrical or optical stimulation, or through internal mechanisms such as genetic modulation and synaptic plasticity. The nervous system, consisting of numerous neurons and synaptic connections [24,25], is responsible for perception, information processing, and the regulation of behavior [26]. For such a system to generate specific neural activities, it must possess a certain degree of controllability that allows effective modulation of its dynamics [27].

During development, controllability undergoes significant changes. In the early larval stages of *C. elegans*, sparse connectivity makes it difficult to exert global regulation. As neurons and synapses increase in number, however, the system gradually becomes more responsive to modulation. When muscles and other effector cells are included in the network analysis, controllability is further enhanced, since these outputs contribute to coordinated regulation of neural and muscular components, thereby supporting more refined behaviors.

Within this framework, global controllability and target controllability represent two complementary but distinct perspectives. Global controllability refers to the ability to drive the entire network from any initial state to any desired state through suitable inputs. This property is critical for understanding network-wide dynamics such as balance maintenance, pattern generation, and systemic responses to stimuli. In higher organisms, it also underpins coordination between brain regions, facilitates complex decision-making, and supports efficient information processing by shaping activity patterns across the whole network.

Target controllability, in contrast, emphasizes regulating specific nodes or outputs without requiring full control of the entire system. This is particularly relevant for motor and sensory functions. For instance, muscle activity can be regulated by a small subset of motor neurons, enabling efficient execution of locomotion or other movements. In *C. elegans*, behaviors such as egg-laying can be achieved by precisely controlling only a limited number of neurons and associated muscles, without the need for global intervention. This focus on key nodes not only reduces computational and biological cost but also has important implications for medical applications, including neural rehabilitation and neuromodulation therapies.

Taken together, global and target controllability illustrate how neural systems balance flexibility with efficiency. In the case of *C. elegans*, examining both perspectives across development provides critical insights into how its nervous system reorganizes to accommodate the increasing demands of maturation, ensuring both whole-network regulation and function-specific control.

Our controllability analysis follows the general framework of network control theory, which quantifies how network topology constrains the ability to steer neural dynamics through external or intrinsic inputs [28]. This approach has been widely applied in brain connectomics to study developmental processes and control energy landscapes.

### 2.3. Implementation Details

All analyses were implemented in MATLAB R2017b (MathWorks Inc., Natick, MA, USA) on a workstation equipped with an Intel Core i7-13700 CPU and 32 GB RAM. The structural controllability analysis followed the classical framework proposed by Liu et al. [11], while the target controllability was evaluated according to the algorithm of Gao et al. [12]. The adjacency matrices for each developmental stage were constructed from publicly available connectome datasets of *C. elegans*, with both hermaphrodite and male versions included. For each connectome, the driver nodes and control paths were computed using custom MATLAB scripts based on maximum matching and linear control theory. Figures were generated with MATLAB’s plot and heatmap functions.

All computational codes and processed data are available in the Data Availability section.

## 3. Results

### 3.1. The Controllability of *C. elegans* Neural Network Across Development

The theoretical framework outlined above provides the foundation for analyzing neural networks in practice. To move beyond abstract formulations, it is essential to investigate how controllability emerges and evolves in real biological systems. *C. elegans* offers a unique opportunity in this regard, as its entire connectome has been reconstructed across all larval stages and into adulthood [16]. This developmental dataset enables a systematic examination of how structural changes in the nervous system influence both global and target controllability. By quantifying these properties throughout development, we can uncover general principles of how neural circuits reorganize to support increasingly complex behaviors, and assess how the balance between whole-network regulation and function-specific control shifts as the organism matures. The connectomes of *C. elegans* across eight developmental stages have been reconstructed by Witvliet et al. [16], and further analyzed to reveal network evolution patterns and asymmetries in degree distributions [29].

#### 3.1.1. *C. elegans* Neural Network Across Development

The nematode *C. elegans* passes through four larval stages—L1, L2, L3, and L4—before reaching adulthood [30]. Across these stages, its nervous system undergoes marked structural expansion. The number of neurons and synapses increases steadily, leading to greater connectivity and progressively refined neural circuits. By the adult stage, the network is fully established, supporting coordinated functions such as locomotion, sensory processing, and behavioral regulation. Thus, the developmental trajectory of *C. elegans* represents a transition from a relatively simple architecture to a mature and functionally sophisticated nervous system.

**Figure 2:**
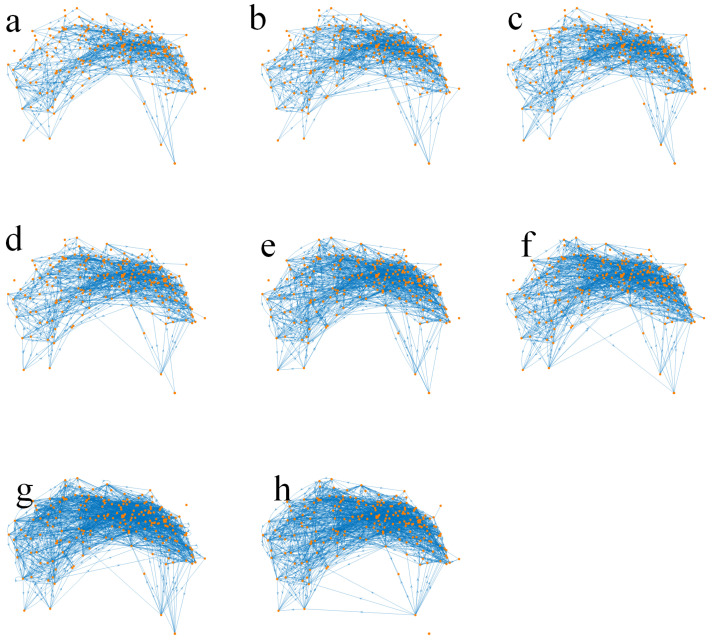
Neural network development.

**Figure 3:**
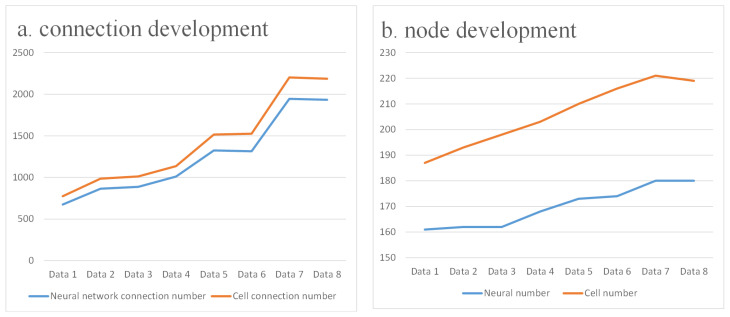
Connection development.

Figure 2 illustrates this developmental process, and the quantitative changes are summarized in Table 1. According to the reconstructions reported by Witvliet et al. [16], the nervous system expands from an early configuration of 161 neurons connected by 675 synapses to a mature network comprising 180 neurons and 1933 synapses (Figure 3). A similar trend is observed at the cellular level, with both cell numbers and connections nearly doubling from larval to adult stages. These anatomical data clearly demonstrate an overall increase in size and density, reflecting the broad structural maturation of the system.

However, such numerical descriptions provide only a superficial view of development. Counting neurons and synapses alone does not reveal how the network’s regulatory capacity or functional organization changes over time. In particular, it remains unclear whether structural growth directly translates into enhanced flexibility, adaptability, or robustness of neural control. To address this gap, control-theoretic approaches offer a deeper perspective. By evaluating measures such as global and target controllability, one can determine whether the expanding connectome actually enables more efficient regulation of neural activity and behavior. This perspective moves beyond static anatomical metrics, highlighting instead how developmental changes in connectivity reshape the dynamical capabilities of the nervous system. In doing so, it provides new insight into the principles by which biological circuits evolve to support increasingly complex behaviors.

#### 3.1.2. Control and Target Control of *C. elegans* Neural Network

Assessing the controllability of the *C. elegans* nervous system at different developmental stages provides critical insight into the robustness and adaptability of its functions. Both global controllability, which reflects the ability of the entire network to transition between states, and target controllability, which focuses on regulating selected outputs, are fundamental to understanding how neural circuits support behavior. In biological systems, sufficient controllability is essential for coordinating locomotion, processing sensory information, and maintaining overall physiological balance. As *C. elegans* develops, the formation of new synapses and the reorganization of existing ones reshape the nervous system’s controllability, thereby influencing its capacity to support increasingly complex and adaptive behaviors.

**Figure 4:**
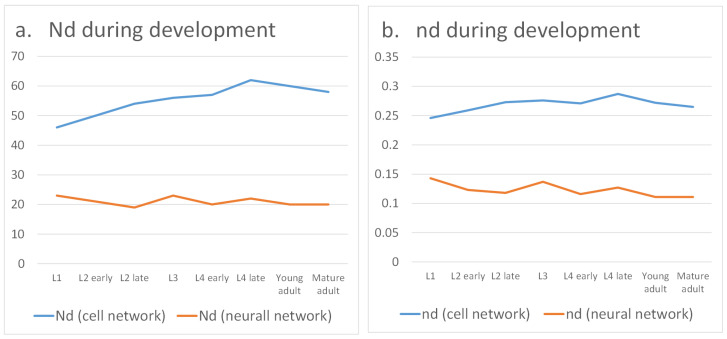
Controllability during development.

The quantitative results of our analysis are summarized in Table 2 and illustrated in Figure 4. At the cell level, which includes both neurons and effector cells such as muscles, the proportion of required driver nodes $N_{d}$ gradually increases with development. This indicates that achieving global controllability across all cells becomes progressively more difficult, suggesting that the worm does not need to coordinate every single cell simultaneously. By contrast, when focusing exclusively on the neural network, the proportion of driver nodes decreases, reflecting improved controllability. This trend highlights that as the nervous system matures, it becomes more efficient at regulating neuronal circuits, thereby enabling refined control of essential behaviors such as locomotion, feeding, and reproduction.

**Figure 5:**
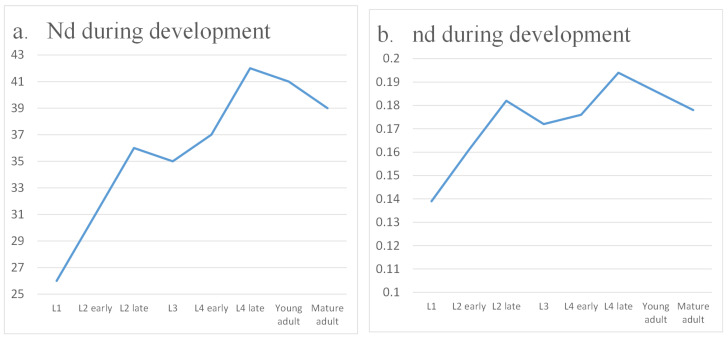
Target controllability during development.

Interestingly, while overall neural controllability improves, the organism’s basic motor output does not require full regulation of every neuron. For locomotion, direct control of muscle cells is often sufficient. This motivates the question of whether *C. elegans* prioritizes muscle-target controllability over neural controllability. Table 3 and Figure 5 present the results of our target control analysis. As expected, fewer control nodes are needed to regulate only muscle outputs than to control the entire network. However, a striking observation emerges: muscle-specific controllability decreases as development progresses. One might anticipate that locomotion, being vital for survival, would remain the highest priority. Instead, once basic motor skills are established during larval stages, the system appears to shift emphasis away from fine-tuned muscle control. This suggests a developmental reallocation of regulatory resources, allowing the nervous system to invest more in additional functions such as egg-laying and higher-order decision-making.

Together, these findings demonstrate a dynamic balance in the control demands of *C. elegans*. While global neural controllability increases to support complex behaviors, target controllability for basic motor functions declines, reflecting a reprioritization of control as the organism matures. This perspective emphasizes that developmental changes in controllability are not simply a matter of increasing network size, but instead reveal functional strategies by which the nervous system allocates resources to meet evolving biological needs.

### 3.2. The Controllability of *C. elegans* Neural Network of Both Sexes

In addition to developmental changes, it is also important to compare the controllability of the *C. elegans* nervous system between sexes. The species exists in two forms—male and hermaphrodite—each with distinct structural and functional features. Male worms possess approximately 375 somatic neurons, with many dedicated to mating behavior, whereas hermaphrodites have about 280 neurons (excluding pharyngeal neurons and CANL/CANR) [6]. Despite this substantial difference in neuronal number, both sexes must sustain effective regulation of their nervous systems to maintain essential behaviors and physiological functions.

To evaluate these differences, we analyzed both global neural controllability and muscle-target controllability (Table 4). The results reveal that, although males have significantly more neurons, their overall controllability is comparable to that of hermaphrodites. Notably, hermaphrodites exhibit slightly higher controllability values in both domains. For the neural network, the fraction of required driver nodes is $n_{d}=0.1794$ in hermaphrodites compared to $n_{d}=0.232$ in males, while for muscle target control, the difference is more modest ($n_{d}=0.0213$ versus 0.0241).

These findings suggest that the structural expansion of the male nervous system, largely associated with mating-specific circuits, does not translate into proportionally improved controllability. On the contrary, hermaphrodites, with fewer neurons, achieve more efficient control. This may reflect different functional priorities: males invest additional neurons into specialized reproductive behaviors, while hermaphrodites maintain a relatively more balanced network architecture that favors overall efficiency. Such differences underscore the value of controllability analysis for understanding how sex-specific neural organization shapes behavioral strategies, offering new insights into the relationship between network structure and functional adaptability.

### 3.3. Influential Neurons Through Development

To further investigate which neurons play a dominant role in maintaining the controllability of the *C. elegans* neural network, we performed an ablation analysis to quantify the contribution of each neuron to global controllability. Specifically, each neuron was removed from the connectome one at a time, and the corresponding decline in controllability was recorded as a measure of its influence. A larger reduction in the overall controllability indicates a more influential neuron in maintaining the functional integrity of the network.

Interestingly, the results revealed a consistent pattern across all developmental stages. The command interneurons, including *AVAL*, *AVAR*, *AVBL*, *AVBR*, *AVEL*, and *AVER*, consistently showed the largest controllability losses upon ablation, highlighting their critical role in coordinating neural dynamics and locomotion-related processes. In contrast, sensory neurons exhibited lower influence scores, while interneurons as a group contributed more to network controllability than motor or sensory neurons. This finding suggests that internal signal integration and coordination, rather than direct sensory input, dominate the controllability structure of the *C. elegans* nervous system.

Moreover, this developmental invariance of key command neurons implies that the architecture of the worm’s nervous system preserves core control centers throughout its maturation. Such structural persistence ensures the stability of fundamental behaviors, while peripheral or specialized circuits can evolve to support higher-level or sex-specific functions. These observations provide an important biological interpretation of controllability: the internal regulatory neurons form the backbone of the neural control hierarchy, enabling adaptive behavior as the organism matures.

## 4. Discussion and Future Perspectives

The present study focuses on the controllability of the *C. elegans* neural network across developmental stages. To place these findings in a broader context, it is important to relate them to ongoing efforts in modeling the nematode’s nervous system.

In recent years, several studies have explored the construction of data-driven or black-box models of *C. elegans*, aiming to approximate neural dynamics and behavioral responses using measured or simulated input–output information [31]. Such models—often implemented through recurrent or graph-based neural network architectures—provide valuable tools for simulating functional behaviors, such as locomotion, sensory processing, and chemotaxis-related information flow [19,20].

These complementary approaches offer a promising direction for integrating structural controllability theory with dynamic simulation. Future work may combine controllability analysis with black-box or reduced-order modeling to build hybrid frameworks that can both explain the structural basis of control and simulate its behavioral consequences. This would allow for more comprehensive studies linking network topology, neural control efficiency, and emergent behaviors across developmental or pathological conditions.

Beyond nematode connectomics, recent large-scale reconstructions such as the complete brain connectome of the *Drosophila* larva have revealed conserved circuit motifs and hierarchical control architectures across species [32]. These findings suggest that principles derived from *C. elegans* controllability analyses could generalize to more complex nervous systems, offering a scalable framework for understanding structural–functional coupling in animal brains.

Overall, our controllability-based framework lays the foundation for such integrative modeling efforts by identifying key neurons and control principles that could guide the design of low-complexity yet biologically meaningful computational models of the *C. elegans* nervous system. Future work may further integrate controllability metrics with experimentally measured neural dynamics, enabling a unified view of structural, functional, and control-based organization in developing nervous systems.

## 5. Conclusions

Controllability and target controllability provide valuable perspectives for linking structure to function in biological neural networks. In the case of *C. elegans*, these measures correspond to the ability to regulate the entire nervous system versus the ability to selectively govern specific outputs such as muscle activity. Our analysis reveals a clear developmental trend: as the worm matures, global neural controllability increases, reflecting a progressively refined and adaptable nervous system. In contrast, muscle-target controllability declines, suggesting that once basic locomotor functions are established, neural resources are increasingly redirected toward more specialized roles such as reproductive behaviors, decision-making, and higher-level regulation.

Another important observation is that only a relatively small number of neurons are required to achieve effective muscle control, while a much larger portion of the nervous system is engaged in coordinating complex neural processes. This raises a fundamental question: is full-scale controllability of all neurons truly necessary for survival, or is targeted controllability for specific functions a more efficient strategy? By emphasizing function-specific control, our results highlight a principle that may extend to larger and more complex nervous systems, where localized or modular regulation could dominate over global control.

Finally, the comparison between hermaphroditic and male *C. elegans* shows that despite males possessing substantially more neurons, their overall controllability is not higher. In fact, hermaphrodites exhibit slightly greater efficiency in both neural and target controllability. This finding suggests that neural network size does not directly translate into controllability, but rather reflects distinct evolutionary priorities and behavioral needs. Such insights provide a deeper understanding of how sex-specific neural architectures balance specialization with efficiency, and they offer a foundation for extending controllability analysis to broader questions in neuroscience and control theory.

## Figures and Tables

**Table 1:** Connection development of *C. elegans*.

	Data 1	Data 2	Data 3	Data 4	Data 5	Data 6	Data 7	Data 8
Neural network connection number	675	865	887	1011	1324	1314	1944	1933
Neural number	161	162	162	168	173	174	180	180
Cell connection number	775	986	1012	1136	1515	1525	2202	2186
Cell number	187	193	198	203	210	216	221	219

**Table 2:** Controllability of *C. elegans* cell and neural networks.

	Data 1	Data 2	Data 3	Data 4	Data 5	Data 6	Data 7	Data 8
$N_{d}$ (cell network)	46	50	54	56	57	62	60	58
$n_{d}$ (cell network)	0.246	0.259	0.273	0.276	0.271	0.287	0.272	0.265
$N_{d}$ (neural network)	23	21	19	23	20	22	20	20
$n_{d}$ (neural network)	0.143	0.123	0.118	0.137	0.116	0.127	0.111	0.111

**Table 3:** Target controllability of *C. elegans* cell network.

	Data 1	Data 2	Data 3	Data 4	Data 5	Data 6	Data 7	Data 8
$N_{d}$	26	31	36	35	37	42	41	39
$n_{d}$	0.139	0.161	0.182	0.172	0.176	0.194	0.186	0.178

**Table 4:** Controllability of *C. elegans* in both sexes.

	Hermaphroditic	Male
$N_{d}$ (Neuro)	72	84
$n_{d}$ (Neuro)	0.1794	0.232
$N_{d}$ (Target)	8	11
$n_{d}$ (Target)	0.0213	0.0241

## Data Availability

The data and code for this study can be found in the [The controllability of *C. elegans* neural network] https://gitee.com/liujianwgx/The-controllability-of-C.-elegans-neural-network.git (accessed on 8 April 2025).

## Abbreviations

The following abbreviation is used in this manuscript:

*C. elegans*	*Caenorhabditis elegans*
